# Supplementary material for: Content-rich biological network constructed by mining PubMed abstracts
Source: BMC Bioinformatics. 2004 Oct 8;5:147. doi: 10.1186/1471-2105-5-147 (PMC528731; doi:10.1186/1471-2105-5-147)
Supplement: Additional File 2 — The original results of the above study (non-essential files are deleted to keep the file size under the limit set by BMC bioinformatics). [file 1471-2105-5-147-S2.bz2 › chilibotAdditionalFile2/dip50/556ID10395669E10916/html/IFNAR2_IFNA1.html]

 


 **IFNAR2** and **IFNA1** 
  
Found 143 abstracts in PubMed, retrieved 50.  
 

 What does Google say? 
 PDF only 
| .edu only 

---

**Interactive relationship** (e.g. stimulation, inhibition, etc)

**Neutral relationship**- The interaction of IFNs with their heterodimeric type I IFN  [ **IFNA1** ]  receptor comprised of IFNAR1 and  **IFNAR2**  is a first step to inducing biological actions.  Ref: 12542398 Biochem J, 2003
- The pleiotropic activity of type I interferons has been attributed to the specific interaction of IFN  [ **IFNA1** ]  with the cell surface receptor components ifnar1 and  **ifnar2** .  Ref: 11698684 Proc Natl Acad Sci U S A, 2001
- The results suggest that the binding energy from interaction of IFN  [ **IFNA1** ]  beta with  **ifnar2**  serves mainly to stabilize the bound IFN  [ **IFNA1** ]  receptor complex.  Ref: 10704203 Biochemistry, 2000
- DO exchange experiments indicate that binding of IFN  [ **IFNA1** ]  alpha2 induces tightening of the N terminal domain of  **IFNAR2**  EC.  Ref: 11888273 Biochemistry, 2002
- Conclusion The combination of IFN  [ **IFNA1** ]  R  [ **IFNAR2** ]  is better as first line treatment than IFN  [ **IFNA1** ]  monotherapy.  Ref: 10613748 Hepatology, 2000
- Liver biopsy specimens from 41 chronic hepatitis C patients subsequently treated with IFN  [ **IFNA1** ]  were immunostained with IFNAR1 and  **IFNAR2**  antibodies, and also analyzed for both receptor subunit mRNA levels, using competitive polymerase chain reaction.  Ref: 12699848 Hepatol Res< MedlineTA>Hepatol Res, 2000
- We are investigating the biophysical basis of IFN  [ **IFNA1** ]  signaling by characterizing the complex of the extra cellular domain of  **ifnar2**   **ifnar2**  EC with IFNs on the level of purified recombinant proteins in vitro.  Ref: 10556041 J Mol Biol, 1999
- Type I interferons IFN  [ **IFNA1** ]  exert pleiotropic activities through binding to two cell surface receptors, ifnar1 and  **ifnar2** .  Ref: 10556041 J Mol Biol, 1999
- The IFN alpha  [ **IFNA1** ]  beta receptor IFNAR 2  [ **IFNAR2** ]  subunit of the IFN  [ **IFNA1** ]  receptor is considered the primary binding chain of the receptor, yet it is clear that both receptor subunits, IFNAR 1 and IFNAR 2  [ **IFNAR2** ] , cooperate in the high affinity binding of IFN  [ **IFNA1** ]  to the receptor complex.  Ref: 10841076 J Interferon Cytokine Res, 2000
- Therefore, our results suggest that soluble Ifnar 2  [ **IFNAR2** ]  is an important regulator of endogenous and systemically administered type I IFN  [ **IFNA1** ] .  Ref: 11154225 Blood, 2001
- Comparing the anti viral potency of the various mutants to the binding affinity towards  **ifnar2**  revealed a proportional correlation between the two, suggesting a rate limiting role of  **ifnar2**  binding in IFN  [ **IFNA1** ]  signaling.  Ref: 10556041 J Mol Biol, 1999
- The concentration of type I interferon receptor  [ **IFNAR2** ]  IFN  [ **IFNA1** ]  Rc in the liver is a crucial factor in determining the efficacy of interferon IFN  [ **IFNA1** ]  therapy in patients with chronic hepatitis C.  Ref: 12518169 J Lab Clin Med, 2003
- The mutants were analyzed for activity in vitro in antiviral and in antiproliferation assays, and for their ability to bind to the type I IFN  [ **IFNA1** ]  ifnar1  **ifnar2**  receptor on Daudi cells and to a soluble  **ifnar2**  fusion protein  **ifnar2**  Fc.  Ref: 10704203 Biochemistry, 2000
- These studies demonstrate the heavy concentration of IFNAR1 and  **IFNAR2**  in cells of the LE and superficial GE, which appear to be the main targets for IFN  [ **IFNA1** ]  tau.  Ref: 12193393 Biol Reprod, 2002
- Type I interferon IFN  [ **IFNA1** ]  receptor has a multichain structure composed of at least two distinct subunits, IFNAR 1 and IFNAR 2  [ **IFNAR2** ] .  Ref: 10505744 J Interferon Cytokine Res, 1999
- Type I IFNs bind to a receptor composed of two transmembrane polypeptides, IFNAR1 and  **IFNAR2** , which belong to the class II cytokine receptor family that also includes the cellular receptors for IFN  [ **IFNA1** ]  gamma, interleukin 10 and coagulation protease factor VII tissue factor.  Ref: 11125298 Eur Cytokine Netw, 2000
- The human type I interferons, IFN alpha  [ **IFNA1** ] , IFN  [ **IFNA1** ]  beta, and IFN  [ **IFNA1** ]  omega, induce somewhat different cellular effects but act through a common receptor complex, IFNAR, composed of subunits IFNAR 1 and IFNAR 2  [ **IFNAR2** ] .  Ref: 11278538 J Biol Chem, 2001
- However, two mAb that bound to sites remote from the IFNAR 2  [ **IFNAR2** ]  binding site on IFN  [ **IFNA1** ]  beta also inhibited IFN  [ **IFNA1** ]  beta IFNAR 2  [ **IFNAR2** ]  complex formation and demonstrated potent neutralizing activity.  Ref: 11747625 J Interferon Cytokine Res, 2001

**Non-interactive relationship** (e.g. studied together, co-existance, homology, etc.)

- Two of these inhibited IFN  [ **IFNA1** ]  beta IFNAR 2  [ **IFNAR2** ]  complex formation, suggesting that steric hindrance of receptor binding constitutes their mechanism of neutralization.  Ref: 11747625 J Interferon Cytokine Res, 2001
- In the present study, we demonstrated that IFN  [ **IFNA1** ]  gamma induced the expression of mRNA for IFNAR 1 and IFNAR 2  [ **IFNAR2** ]  in a human hepatoma cell line, HepG2 cells.  Ref: 10505744 J Interferon Cytokine Res, 1999
- Surprisingly, using primary thymocytes from Ifnar 2  [ **IFNAR2** ]  mice, recombinant muIfnar 2a formed a complex with IFN alpha  [ **IFNA1** ]  or beta and muIfnar 1 at the cell surface and transmitted an antiproliferative signal.  Ref: 11154225 Blood, 2001
- KM12L4 IFN alpha  [ **IFNA1** ]  sensitive or KM12L4 IFN  [ **IFNA1** ]  R  [ **IFNAR2** ]  IFN alpha  [ **IFNA1** ]  resistant cells were injected into the spleen of nude mice.  Ref: 11420751 Neoplasia< MedlineTA>Neoplasia, 2001
- Tolerability was good only 11 in IFN  [ **IFNA1** ]  R  [ **IFNAR2** ]  and 8 patients IFN  [ **IFNA1** ]  L group respectively had to stop therapy due to side effects.  Ref: 11945144 Curr Pharm Des, 2002
- These results show that the neutralization of type I IFN  [ **IFNA1** ]  biologic actions by anti IFNAR 2  [ **IFNAR2** ]  mAb cannot be entirely explained by inhibition of Jak Stat tyrosine phosphorylation.  Ref: 11096454 J Interferon Cytokine Res, 2000
- The mutual binding sites on IFN alpha  [ **IFNA1** ]  2 and  **ifnar2**  predicted from the model showed an almost complete superposition with the ones determined from mutagenesis studies.  Ref: 11698684 Proc Natl Acad Sci U S A, 2001
- To assess whether hepatitis C virus infection HCV affects type I interferon IFN  [ **IFNA1** ]  receptor expression, the expression levels of two subunits of this receptor, IFNAR1 and  **IFNAR2** , in peripheral blood mononuclear cells PBMCs were determined by flow cytometric assay in 68 anti HCV antibody positive patients 47 positive for HCV RNA and 21 negative for HCV RNA and 14 healthy controls.  Ref: 12787799 Hepatol Res< MedlineTA>Hepatol Res, 2001
- The production of IFN  [ **IFNA1** ]  r  [ **IFNAR2** ]  IL 4 by CD4 T cells in PBMC was determined by fluorescence activated call sorting FACS analysis and fluorescence quantitative PCR assay for quantification of HBV DNA.  Ref: 11509144 Zhonghua Gan Zang Bing Za Zhi, 2001
- a sustained response was seen in 16% of IFN  [ **IFNA1** ]  R  [ **IFNAR2** ]  and in 19% of IFN  [ **IFNA1** ]  L patients.  Ref: 11945144 Curr Pharm Des, 2002
- Analysis of these deviations maps the IFN alpha  [ **IFNA1** ]  2 binding site upon  **IFNAR2**  EC to a contiguous surface on the N terminal domain, including the S3 S4 loop, the S5 S6 loop and S6 beta strand, and the S7 beta strand and the hinge region.  Ref: 11888273 Biochemistry, 2002
- 253J B V IFN  [ **IFNA1** ]  R  [ **IFNAR2** ]  cells resistant to antiproliferative effects of IFN alpha  [ **IFNA1** ]  or IFN  [ **IFNA1** ]  beta were implanted into the bladder wall of nude mice.  Ref: 10537335 Clin Cancer Res, 1999
- Expression of Hu IFN  [ **IFNA1** ]  alphaR2 chain of type I interferon receptor  [ **IFNAR2** ]  in human hepatocellular carcinoma and non cancerous tissues.  Ref: 11078820 Int J Mol Med, 2000
- In this report, we have used mice that are deficient in components of the early defence system, the common type I interferon IFN  [ **IFNA1** ]  receptor IFN  [ **IFNA1** ]  R  [ **IFNAR2** ] , the transcriptional activator IRF 1, and the inducible nitric oxide synthase, to investigate the contribution of these mechanisms to control of MHV 68 infection.  Ref: 10497103 Virology, 1999
- Their relationship to the outcome of antiviral therapy for chronic HCV infection was studied in 49 white patients who had a virologically sustained response SR and in 55 white nonresponders NR to a combination of interferon alfa 2b and ribavirin IFN  [ **IFNA1** ]  R  [ **IFNAR2** ] .  Ref: 11230752 Hepatology, 2001
- Upregulation of type I interferon receptor  [ **IFNAR2** ]  by IFN  [ **IFNA1** ]  gamma.  Ref: 10505744 J Interferon Cytokine Res, 1999
- To determine whether phosphorylation of tyrosine residues in the IFN  [ **IFNA1** ]  receptor is required for activation of the PI 3 kinase, we performed studies using mouse L929 fibroblasts transfected with mutated human IFNAR1 and or  **IFNAR2**  subunits of the Type I IFN  [ **IFNA1** ]  receptor, lacking tyrosine phosphorylation sites.  Ref: 10733921 Biochem Biophys Res Commun, 2000
- The 117.7 mAb, representative of this class of receptor nonblocking mAb, induced hyper tyrosine phosphorylation of IFNAR 2  [ **IFNAR2** ]  in the presence of IFN alpha  [ **IFNA1** ]  beta but did not inhibit IFN alpha  [ **IFNA1** ]  beta induced Jak Stat tyrosine phosphorylation and ISGF3 complex formation.  Ref: 11096454 J Interferon Cytokine Res, 2000
- Alternative splicing leads to the expression of multiple isoforms of the subunits IFNAR1 and  **IFNAR2**  of the type I IFN  [ **IFNA1** ]  receptor.  Ref: 11344274 Proc Natl Acad Sci U S A, 2001
- The structure of the  **IFNAR2**  IFN  [ **IFNA1** ]  binding ectodomain  **IFNAR2**  EC, the first helical cytokine receptor structure determined in solution, reveals the molecular basis for IFN  [ **IFNA1** ]  binding.  Ref: 12842042 Structure Camb , 2003
- There was a significant advantage for IFN  [ **IFNA1** ]  R  [ **IFNAR2** ]  in comparison with IFN  [ **IFNA1** ]  alone whatever the combination of factors.  Ref: 10613748 Hepatology, 2000
- Patients were randomised to receive either IFN  [ **IFNA1** ]  R  [ **IFNAR2** ]  or IFN  [ **IFNA1** ]  L.  Ref: 11945144 Curr Pharm Des, 2002
- Randomized trials have shown the enhancement of efficacy with interferon alfa 2b and ribavirin IFN  [ **IFNA1** ]  R  [ **IFNAR2** ]  in comparison with interferon monotherapy IFN  [ **IFNA1** ]  as first line treatment of chronic hepatitis C.  Ref: 10613748 Hepatology, 2000
- The neutralization of type I IFN  [ **IFNA1** ]  biologic actions by anti IFNAR 2  [ **IFNAR2** ]  monoclonal antibodies is not entirely due to inhibition of Jak Stat tyrosine phosphorylation.  Ref: 11096454 J Interferon Cytokine Res, 2000
- RESULTS The maximum induction of NOS activity was observed in hepatocytes treated by LPS in combination with IFN  [ **IFNA1** ]  r  [ **IFNAR2** ] , TNF alpha and IL 1beta or IL 6.  Ref: 11412395 Zhonghua Gan Zang Bing Za Zhi, 2001
- Here, we quantitatively mapped the complete binding region of  **ifnar2**  on interferon IFN  [ **IFNA1** ]  alpha2 by 35 individual mutations to alanine and isosteric residues.  Ref: 10984492 J Biol Chem, 2000
- RESULTS Single rectal administration of TNBS developed significant colitis in IFN  [ **IFNA1** ]  R  [ **IFNAR2** ]  mice and anti IFN  [ **IFNA1** ]  gamma mAb pretreated mice, as well as control wild type mice.  Ref: 12702051 J Gastroenterol Hepatol< MedlineTA>J Gastroenterol Hepatol, 2000
- IFN  [ **IFNA1** ]  r  [ **IFNAR2** ]  IL 4 expression in peripheral blood mononuclear cells of the patients with chronic severe hepatitis < ArticleTitle>.  Ref: 11509144 Zhonghua Gan Zang Bing Za Zhi, 2001
- The subunits are referred to as IFN  [ **IFNA1** ]  AR1 IFN alpha  [ **IFNA1** ]  receptor and  **IFNAR2**  IFN alpha  [ **IFNA1** ]  beta receptor.  Ref: 11494550 Nippon Rinsho, 2001
- Local injection of TGF beta 1 and IFN  [ **IFNA1** ]  r  [ **IFNAR2** ]  can promote and inhibit the formation of excessive dermal scarring respectively.  Ref: 11767702 Zhonghua Zheng Xing Wai Ke Za Zhi, 2001
- Pulmonary histopathology was only aggravated in IFN  [ **IFNA1** ]  R  [ **IFNAR2** ]  mice, characterised by eosinophilic influx around the bronchioles.  Ref: 11857536 J Med Virol, 2002
- Here, we present a detailed mutational study on the functional epitopes on both IFN  [ **IFNA1** ]  and  **ifnar2** .  Ref: 10556041 J Mol Biol, 1999
- Multistart minimization and Monte Carlo minimization docking of IFN alpha  [ **IFNA1** ]  2 onto  **ifnar2**  converged to a well defined average structure, with the five distance constraints being satisfied.  Ref: 11698684 Proc Natl Acad Sci U S A, 2001
- A proposal is made that the IFN  [ **IFNA1** ]  receptor, with its subunits IFNAR 1 and IFNAR 2  [ **IFNAR2** ] , presents two separate ligand binding sites, and this double structure is both necessary and sufficient to ensure that the different IFN  [ **IFNA1** ]  are recognized and can act selectively.  Ref: 10547147 J Interferon Cytokine Res, 1999
- Co infection was associated with lower  **IFNAR2**  mRNA levels, higher levels of serum HCV RNA, and a poor IFN  [ **IFNA1** ]  response, regardless of the HCV genotype.  Ref: 11170061 J Med Virol, 2001
- Both production of IFN alpha  [ **IFNA1** ]  and killing activity of NK resumed to the control level on the 6th day, production of IFN  [ **IFNA1** ]  r  [ **IFNAR2** ]  and CD25 were significantly lowered P <.  Ref: 11765771 Space Med Med Eng Beijing , 1999
- Here we describe two transcripts representing extracellular forms of ovine IFNAR1 and show that soluble extracellular forms of both  **IFNAR2**  and IFNAR1, prepared in recombinant form in Escherichia coli, have antiviral AV activity in the absence of IFN  [ **IFNA1** ] .  Ref: 11344274 Proc Natl Acad Sci U S A, 2001
- In conclusion, homozygosity for 592A, 819T or the extended haplotype 108bp 2575T 2763C 1082A 819T 592A is associated with SR to IFN  [ **IFNA1** ]  R  [ **IFNAR2** ] .  Ref: 11230752 Hepatology, 2001
- Anti IFNAR 2  [ **IFNAR2** ]  mAb 117. 5. 3.2, and 51.44 neutralized type I IFN  [ **IFNA1** ]  mediated antiviral, antiproliferative, and major histocompatibility complex MHC class I upregulation functions.  Ref: 11096454 J Interferon Cytokine Res, 2000
- Here the extracellular domain of  **IFNAR2** , a soluble 25 kDa IFN  [ **IFNA1** ]  binding polypeptide, and its complex with IFN alpha  [ **IFNA1** ]  2 were studied using multidimensional NMR.  Ref: 11888273 Biochemistry, 2002
- Abolition of binding to  **ifnar2**  Fc for mutants A2, AB1, AB2, and E established that the  **ifnar2**  binding site on IFN  [ **IFNA1** ]  beta comprises parts of the A helix, the AB loop, and the E helix.  Ref: 10704203 Biochemistry, 2000
- The aim of this study was to answer this question by analyzing the data from 44 naive patients included in trials that compared 24 or 48 week IFN  [ **IFNA1** ]  R  [ **IFNAR2** ]  treatment.  Ref: 10613748 Hepatology, 2000
- Type I interferon receptor  [ **IFNAR2** ]  blocking antibody reversed all PDC effects, and recombinant IFN alpha  [ **IFNA1** ] , beta or omega replicated all effects, except reduced CD40.  Ref: 11745405 Eur J Immunol, 2001
- The focus of this study is to determine both mRNA and protein expression levels of the type I interferon IFN  [ **IFNA1** ]  receptor subunits, IFNalpha receptor IFNAR1 and IFNalpha beta receptor  **IFNAR2** , in the liver, and to assess which quantification method is most useful in predicting response to IFN  [ **IFNA1** ]  in chronic hepatitis C patients.  Ref: 12699848 Hepatol Res< MedlineTA>Hepatol Res, 2001
- In this study, the structure of the IFN alpha  [ **IFNA1** ]  2  **ifnar2**  complex was generated with a docking procedure, using nuclear Overhauser effect like distance constraints obtained from double mutant cycle experiments.  Ref: 11698684 Proc Natl Acad Sci U S A, 2001
- The effects of LPS associated with IFN  [ **IFNA1** ]  r  [ **IFNAR2** ] , TNF alpha and IL 1beta or IL 6 on NOS activity, cGMP, and NO 2 NO 3 were observed in hepatocytes, respectively.  Ref: 11412395 Zhonghua Gan Zang Bing Za Zhi, 2001
- The two types of IFN  [ **IFNA1** ]  showed a comparable efficacy an end of therapy response was observed in 34% of IFN  [ **IFNA1** ]  R  [ **IFNAR2** ]  and 30% of IFN  [ **IFNA1** ]  L patients.  Ref: 11945144 Curr Pharm Des, 2002
- 16 chromosomes and one copy each of disabled IFN  [ **IFNA1** ]  gamma receptor IFNGR and IFN alpha  [ **IFNA1** ]  beta receptor IFNAR 2  [ **IFNAR2** ]  component genes.  Ref: 10714556 J Interferon Cytokine Res, 2000
- A panel of monoclonal antibodies mAb derived against human interferon alpha beta receptor 2  [ **IFNAR2** ]  IFNAR 2  [ **IFNAR2** ]  was evaluated for their ability to antagonize the biologic effects of type 1 interferons IFN  [ **IFNA1** ]  alpha1, IFN  [ **IFNA1** ]  alpha2a, and IFN  [ **IFNA1** ]  beta.  Ref: 11096454 J Interferon Cytokine Res, 2000
- We constructed chimeric receptors wherein the extracellular domain of the erythropoietin receptor EpoR was fused to the transmembrane and intracellular domains of the interferon IFN  [ **IFNA1** ]  type I receptor subunits, IFNaR1 or  **IFNaR2**  2.  Ref: 10574956 J Biol Chem, 1999
- We run a randomised controlled trial with the aim to compare tolerability and efficacy of two different types of IFN  [ **IFNA1** ]  recombinant alpha 2b interferon IFN  [ **IFNA1** ]  R  [ **IFNAR2** ]  and leukocyte alpha n 3 interferon IFN  [ **IFNA1** ]  L at the same dosage of 3 MU subcutaneously thrice weekly for one year.  Ref: 11945144 Curr Pharm Des, 2002
- The intrahepatic mRNA levels of  **IFNAR2** , one of the two subunits of the type I IFN  [ **IFNA1** ]  receptor, were quantified and compared with both the prevalence of HBV DNA and the hepatitis C virus HCV genotype in 45 patients with chronic hepatitis C, who were negative for hepatitis B surface antigen.  Ref: 11170061 J Med Virol, 2001
- Furthermore, adult Stat1, IFN  [ **IFNA1** ]  gamma, and IFN alpha  [ **IFNA1** ]  beta receptor IFNAR 2  [ **IFNAR2** ]  mice infected with rotavirus or vaccinated with VP6 vaccine and adjuvant were fully protected against rotavirus shedding following a subsequent challenge with rotavirus.  Ref: 12716489 J Interferon Cytokine Res, 2003
